# Improvement of the reversible unilateral ureteral obstruction model

**DOI:** 10.1080/0886022X.2025.2454721

**Published:** 2025-03-17

**Authors:** Tungchiang Chen, Zhihua Zhang, Caiyong Lai

**Affiliations:** ^a^ Department of Urology, The First Affiliated Hospital of Jinan University, Guangzhou, China; ^b^ Department of Urology, The Sixth Affiliated Hospital of Jinan University, Dongguan, China; ^c^ Department of Pediatric Surgery, Huizhou Central People’s Hospital, Huizhou, China

**Keywords:** Dynamic renal scintigraphy, glomerular filtration rate, hydronephrosis, MRI, obstructive nephropathy, reversible unilateral ureteral obstruction model

## Abstract

The reversible unilateral ureteral obstruction (RUUO) model is pivotal for studying obstructive nephropathy (ON) but has limitations, including procedure complexity and inconsistent recanalization success. We developed a simpler, reliable, and efficient RUUO model, and utilized advanced auxiliary examination methods to assess hydronephrosis and renal function changes, providing evidence for procedural success. Male Sprague–Dawley rats were divided into control and experimental groups. Baseline data on glomerular filtration rate (GFR) and magnetic resonance imaging (MRI) were obtained from the control group. The experimental group was subdivided based on obstruction durations of 3, 7, 10, and 14 days. Unilateral ureteral obstruction models were created followed by obstruction release to establish the RUUO models. Dynamic renal scintigraphy with single-photon emission computed tomography was used to measure left kidney GFR pre-recanalization and on day 7 and 14 post-recanalization. MRI was used to evaluate hydronephrosis resolution. Key surgical modifications included complete removal of ligated ureter segments and wider ureter–bladder anastomosis, improving consistency and 93.35% recanalization success rate. MRI and ^99m^Tc-DTPA dynamic renal scintigraphy indicated varying degrees of renal functional recovery. The 3-day obstruction group showed near-complete restoration within 1 week of recanalization. Conversely, extended obstruction durations significantly impaired recovery. The 14-day group demonstrated marked functional decline due to progressive renal fibrosis observed at 2 weeks post-recanalization. The optimized model offers simplified surgical techniques, enhanced recanalization success, and high reproducibility. These findings highlight the importance of early recanalization in preserving renal function, and provide a robust framework for future research on ON, including therapeutic strategies.

## Introduction

The prevalence of obstructive nephropathy (ON) has been rising steadily, contributing to an increased incidence of end-stage renal disease (ESRD) worldwide [1]. ON is among the most common urinary tract disorders, ranking fourth in incidence among men and sixth among women. Additionally, ON accounts for approximately 2% of ESRD cases [2].

The unilateral ureteral obstruction (UUO) model has been a cornerstone for studying the molecular and cellular mechanisms underlying renal interstitial fibrosis [3–5]. It is favored for its straightforward methodology, high success rate, and reproducibility. However, its inability to simulate renal repair and functional recovery limits its utility.

The reversible UUO (RUUO) model has gained recognition for its ability to evaluate renal function recovery following obstruction release. This model also enables analysis of renal functional changes at varying recanalization times.

At present, three primary methods are commonly used to establish the RUUO model: plastic tube intervention [6–9], microvascular clamping [10,11], and microscopic surgery [12–14]. Among these, the first two methods are simple and repeatable; however, they are characterized by unstable outcomes and relatively low recanalization success rates. In contrast, microscopic surgery offers higher precision but presents significant challenges, including complex procedures, lengthy operation times, and extended training cycles, which limits its practicality and widespread application. To address these limitations, this study aimed to develop a novel and improved approach to the RUUO model. The proposed method focuses on resolving issues such as incomplete ureteral recanalization, low recanalization success rate, and surgical inconsistencies. By optimizing the surgical techniques, this model provides a simpler, more reliable, and highly reproducible alternative, making it accessible and effective for researchers in this field.

## Materials and methods

### Animals

Male Sprague–Dawley rats (specific pathogen-free [SPF] grade), aged 8–10 weeks, and weighing 300 ± 50 g, were obtained from Hunan SJA Laboratory Animal Co., Ltd (License No.: SCXK [Xiang] 2019–0004). The animals were housed at the Jinan University First Affiliated Hospital Experimental Animal Center (Use License No.: SYXK [Yue] 2017–0174). After a 1-week acclimation period, the rats were randomly assigned to experimental groups using computer-generated random sequences. To minimize potential confounders, the animals were randomly assigned to cages, and the sequence of treatments and measurements was randomized. Furthermore, the cages’ positions within the facility were rotated weekly to mitigate potential environmental influences.

### Experimental reagents

3% pentobarbital sodium (30 mg/kg), 80,000 U/mL of penicillin sodium, and Technetium-99m Diethylenetriamine Pentaacetic Acid (^99m^Tc-DTPA) (37 MBq; 1 mCi).

### Experimental equipment

Surgical microscope, micro tweezers, micro shear, micro needle-holding forceps, micro hemostatic forceps, surgical scissors, deep retractor, sutures (4–0 and 10–0, round needle), single-photon emission computed tomography (SPECT; Optima NM/CT640, GE), MRI scanner (Discovery MR750, 3.0 T, GE).

### Grouping of the animals

**Figure 1.**
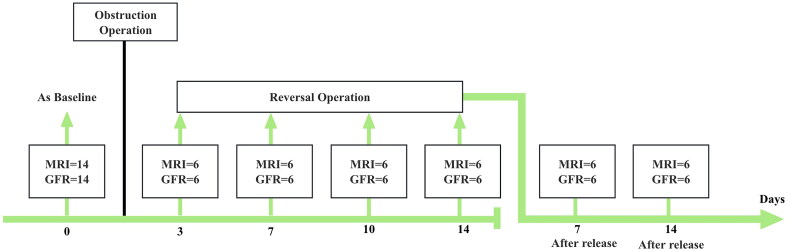
Scheme of the experimental imaging protocol used in this study.

This study used 50 SPF-grade male Sprague–Dawley rats (300 ± 50 g). Fourteen rats were assigned to the blank group to measure preoperative glomerular filtration rate (GFR) values and establish baseline controls using MRI results. The remaining 36 rats formed the experimental group and were randomly divided into four subgroups, each corresponding to a different obstruction duration. Over 3 months, 36 left upper urinary tract UUO models were established. Subsequently, obstruction was released at 3, 7, 10, and 14 days post-obstruction to create the RUUO models. Hydronephrosis and renal function were evaluated using MRI and SPECT before recanalization and at 7 and 14 days post-recanalization (Figure 1).

### Procedure for establishing the UUO model

**Figure 2.**
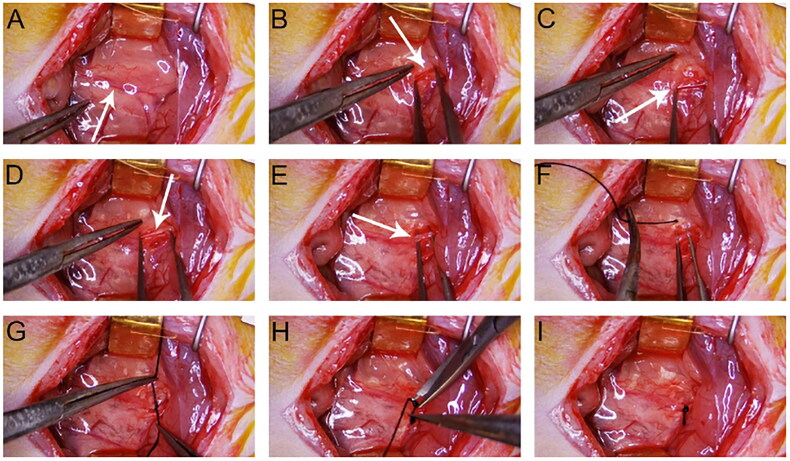
UUO surgery pictures. (A) Normal left ureter (indicated by the arrow). (B) Incising the surface peritoneum along the distal end of the ureter. (C–E) Dissecting the distal ureter outside the ureteral sheath. (F–G) Passing 4-0 silk suture beneath the ureter and tying a knot. (H–I) Trimming the excess suture tails with micro scissors to complete the ureteral ligation.

**Figure 3.**
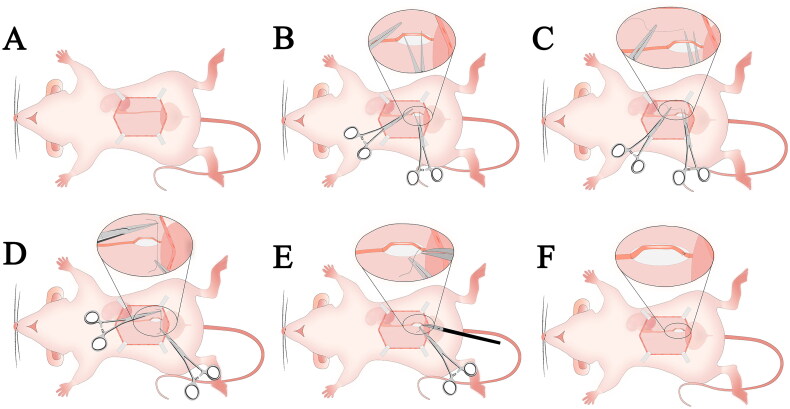
UUO-schematic diagram. (A) Incising the abdominal muscle along the linea alba, creating an approximately 3.5 cm incision to expose the left ureter. (B) Dissecting the distal segment of the ureter outside the ureteral sheath. (C–D) Passing a 4-0 silk suture beneath the ureter and tying a knot. (E–F) Trimming the excess suture tails to complete the ligation of the left ureter.

Anesthesia was induced using 3% pentobarbital sodium (30 mg/kg) administered intraperitoneally. After depilating the abdomen, securing the limbs and teeth, and disinfecting the area with iodophor, a 3.5- to 4.0-cm median abdominal incision was made. The left kidney and ureter were exposed by displacing the intestines. Under 8–10× magnification, the lower segment of the left ureter was carefully isolated and ligated near the bladder using 4–0 silk sutures. Warm saline (5 mL) was injected intraperitoneally, and the abdominal wall was closed in layers. Postoperative care included intramuscular injectors of penicillin (80,000 units) for 3 days (Figures 2 and 3).

### Procedure for establishing the RUUO model

Intraperitoneal administration of 3% pentobarbital sodium (30 mg/kg) was performed to anesthetize the rats. The abdominal area was depilated, and the limbs and teeth were secured. The surgical site was disinfected using an iodophor. The original abdominal surgical incision was reopened, cutting through the skin, subcutaneous tissue, and peritoneum. Upon entering the abdominal cavity, adhesions were carefully released, and the abdominal contents were exposed using a deep retractor to identify the left kidney and ureter. The left intestinal tube was moved to the right, and gauze was placed to minimize fluid loss.

Using 8–10× magnification, the black knot near the ligated left ureter was removed with micro hemostatic forceps, tweezers, and scissors, preserving approximately 1.5 cm of the dilated proximal ureter. A 2- to 3-mm longitudinal incision was made on the left bladder wall to confirm urine outflow. The dilated ureter was then cut 2–3 mm above the ligation knot.

#### Ureteral implantation

End-to-end ureterovesical anastomosis was conducted using a 10–0 microsurgical suture. The initial stitch involved inserting the needle from outside to inside on the left side of the bladder incision and from inside to outside on the left side of the ureteral opening. A 5-cm-long suture tail was left for subsequent bladder rotation. The anterior wall of the ureter–bladder junction was sutured intermittently. The anastomosis site was rotated 180° using the suture tail, facilitating suturing of the posterior wall. The bladder was then rotated back to its physiological position, and the suture tail was trimmed to finalize the anastomosis.

**Figure 4.**
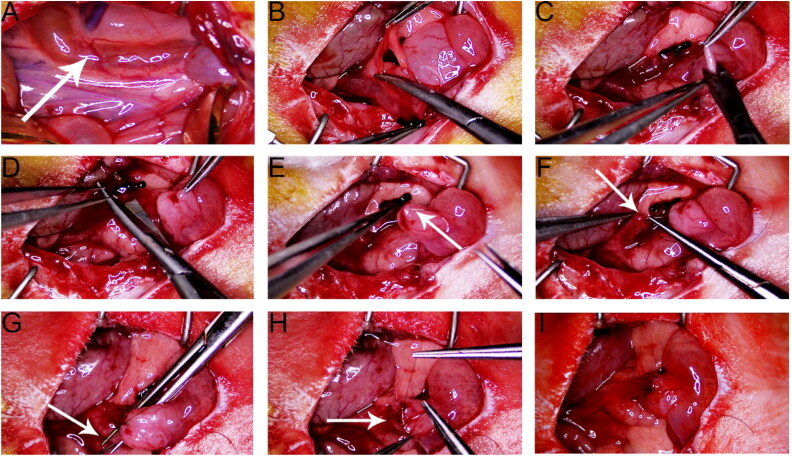
RUUO surgery pictures. (A) Dilated ureter post-obstruction (indicated by the arrow). (B) Releasing the ureter and trimming surrounding adipose tissue. (C) Incising the left wall at the base of the bladder. (D) Cutting the dilated ureter at the black ligation knot. (E) Inserting the first suture through the bladder incision as a fixation point (indicated by the arrow) while leaving approximately 5 cm of suture tail. (F) Performing end-to-end anastomosis of the ureter and the anterior bladder wall. (G) Using the 5 cm suture tail to rotate the bladder. (H) Performing end-to-end anastomosis of the ureter and the posterior bladder wall. (I) Using the 5 cm suture tail again to rotate the bladder back to its physiological position.

**Figure 5.**
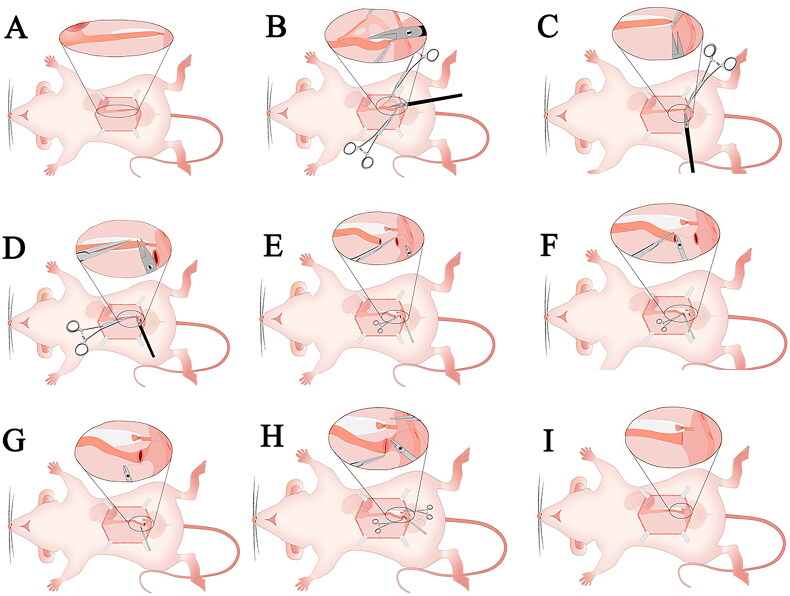
RUUO-schematic diagram. (A) Incising the abdominal muscle along the linea alba, creating an approximately 3.5 cm incision to expose the dilated left ureter. (B) Separating the ureter and trimming the surrounding adipose tissue. (C) Incising the left wall at the base of the bladder. (D) Cutting the ligated segment of the left ureter. (E) Inserting the first suture at the left side of the bladder incision as a fixation point, leaving approximately 5 cm of suture tail. (F) Performing end-to-end anastomosis of the ureter to the anterior bladder wall. (G) Using the 5 cm suture tail to rotate the ureter-bladder anastomosis site. (H) Performing end-to-end anastomosis of the ureter to the posterior bladder wall. (I) Using the 5 cm suture tail again to rotate the bladder back to its physiological position.

The abdominal cavity was flushed with warm saline, and the surgical site was inspected to ensure knot security and the absence of bleeding. The intestines were repositioned, and the abdominal wall was closed in layers. The peritoneum and abdominal white lines were sutured with 4–0 silk thread, and the skin was closed similarly. Postoperative care included intramuscular injections of penicillin (80,000 units) for 3 days (Figures 4 and 5).

### Instrumentation and conditions for MRI

Magnetic resonance imaging was performed using a 3.0-T MRI scanner (MR750, GE, USA) equipped with a 5-cm inner-diameter bowl joint coil. The abdomen of each rat was wrapped with a rubber band to minimize breathing-induced movement. The scanning conditions were as follows: repetition time/echo time (TR/TE), 2500 ms/85 ms; matrix, 288 × 44; field of view, 8.0 cm; slice thickness, 2.9 mm; slice spacing, 0.0 mm; bandwidth, ±15.63 kHz; signal average, 2; total acquisition time, 2 min 25 s; and number of excitations (NEX), 4. MRI data were transferred to an offline workstation (Sun Advantage Workstation 4.5, GE, USA). Morphological and functional changes in the renal parenchyma were assessed using T2-weighted imaging (T_2_-WI) by two radiologists with 15 and 3 years of experience in urogenital imaging.

### Instrument and conditions for dynamic renal scintigraphy

Dynamic renal scintigraphy was conducted using SPECT (Optima NM/CT640, GE, USA) with a low-energy high-resolution collimator. The radioactivity count of a full syringe was measured for 10 s before injection. The drug center point was located in the center of the probe. After measurement, the rats were positioned on their backs on their imaging bed, with the probe placed behind them, facing upward to the target dual kidneys. Images were collected immediately following intravenous bolus administration of 1 mL of ^99m^Tc-DTPA (37 MBq; 1 mCi) *via* a venous line, followed by a 0.9% saline flush.

The scanning conditions were as follows: a dynamic matrix of 64 × 64, amplified threefold; 1 s/frame for the first 60 s, followed by 30 s/frame for the subsequent 18 frames, for a total scan duration of 10 min. After scanning, the syringe’s radioactivity was measured for 10 s (empty syringe).

**Figure 6.**
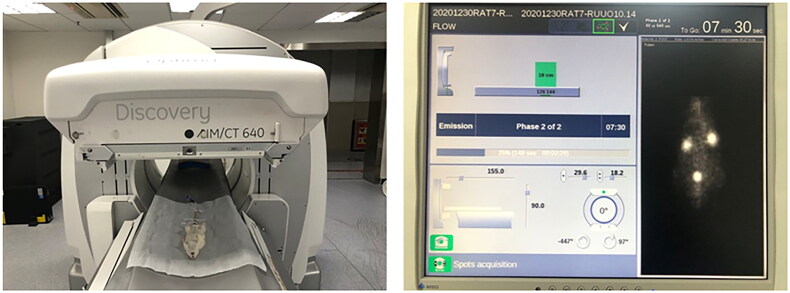
SPECT-procedure for renal function assessment.

An experienced nuclear medicine physician interpreted the scintigraphy images, which were further analyzed using the renal analysis program on the Xeleris workstation. The rats’ age, height, and weight were recorded. Renal and background regions of interest (ROIs) were defined from the kidney and infrarenal background areas. Camera-based GFR, time to maximum concentration (Tmax), and renogram curves were generated and evaluated (Figure 6).

### Statistical analysis

Baseline data from the control and surgical groups were statistically analyzed using SPSS 20.0. Non-normally distributed renal function measurements were expressed as a median and interquartile range, 50 (P25, P75). Repeated measures analysis of variance was used for the analysis, and intergroup comparisons of left kidney GFR values were conducted to assess renal function impairment and recovery. Statistical significance was set at *p* < 0.05. Graphs were generated using GraphPad Prism 10 software.

## Results

### Success rate of obstruction release

On Days 7 and 14 post-RUUO, recanalization success was confirmed using MRI. Obstruction was deemed successful when significant left kidney hydronephrosis was observed on Days 3, 7, 10, and 14 post-UU. Recanalization success was confirmed by a reduction in hydronephrosis on MRI at 7 and 14 days post-RUUO. Two rats in the RUUO group showed incomplete recanalization—one failure occurred in the 10-day group, and another in the 14-day group. An autopsy revealed ischemic necrosis of the ureteral wall in these cases. Consequently, these rats were excluded from the analysis, yielding a recanalization success rate of 93.35% for the modified RUUO model.

### The improved RUUO model

**Figure 7.**
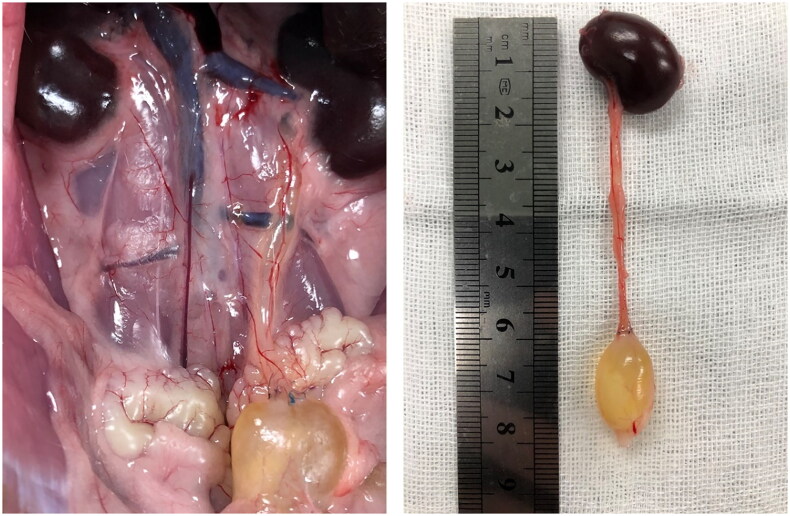
RUUO surgical model specimen.

The swollen ureter and filled bladder within the rat’s abdominal cavity are visible after ureteral recanalization. Even following specimen excision, the surgical site maintains an intact RUUO model (Figure 7).

### MRI changes of the kidney

**Figure 8.**
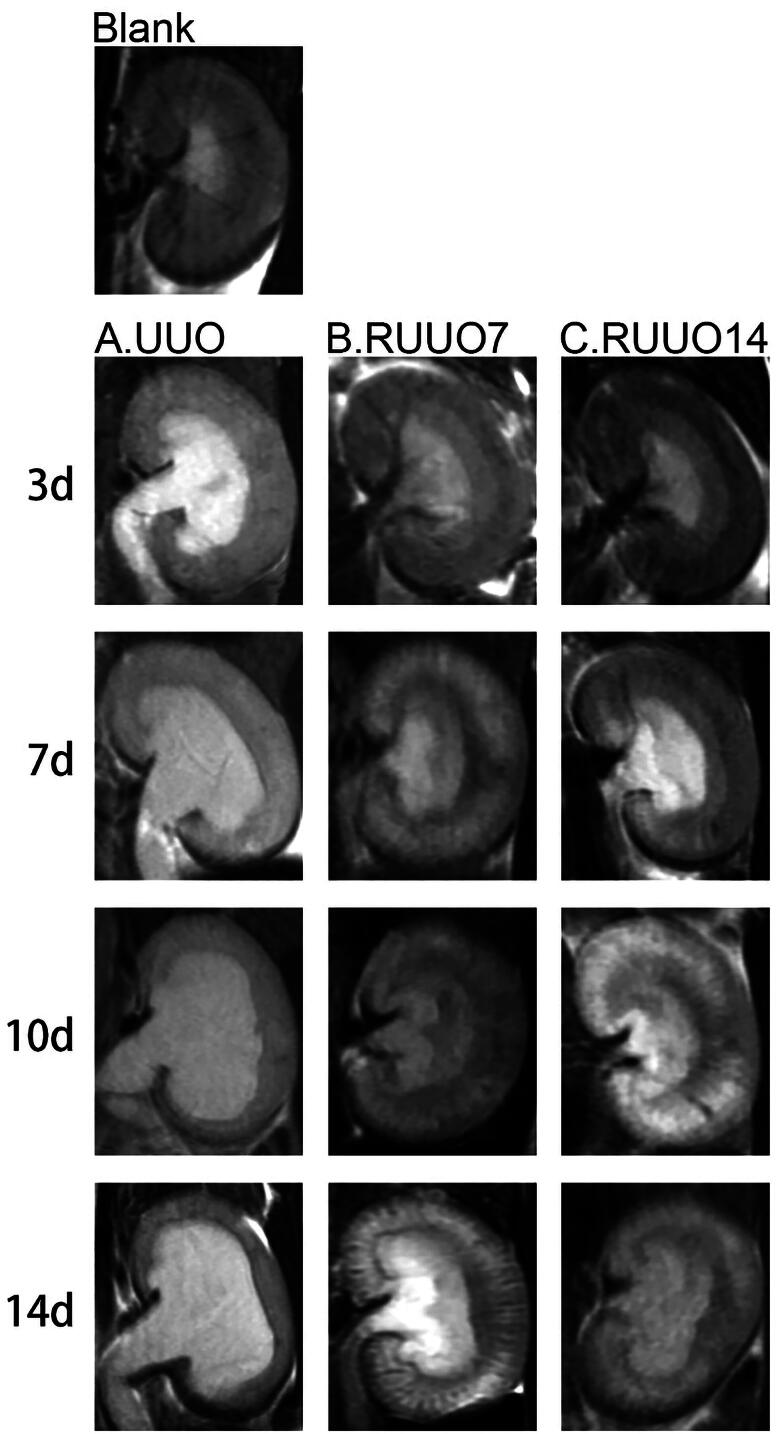
Changes in hydronephrosis after obstruction and recanalization. Renal pelvic dilation increased proportionally with the duration of obstruction. After 7 days of recanalization, renal pelvis dilation and hydronephrosis relief varies according to the initial obstruction duration. The 3-day obstruction group showed the quickest recovery, while the 14-day group exhibited the lowest. By Day 14 post-recanalization, all groups demonstrated significant improvement in hydronephrosis and renal pelvis recovery. These findings highlight the efficacy of the modified recanalization model in alleviating hydronephrosis.

Blank: Normal left kidney; no dilation of the renal pelvis; no hydronephrosis. 3d: On Day 3 post-obstruction, the left kidney exhibited hydronephrosis and renal pelvis dilation. By Day 7 post-recanalization, the hydronephrosis had decreased, the renal pelvis was smaller, and the medulla and cortex were distinguishable. By Day 14 post-recanalization, there was minimal fluid retention, the renal pelvis was near normal in size, and the medulla and cortex layers were distinctly visible. 7d: On Day 7 post-obstruction, the left kidney showed moderate hydronephrosis with significant renal pelvis dilation. By Day 7 post-recanalization, the hydronephrosis had reduced, the renal pelvis was smaller, and the distinction between the medulla and cortex was slightly blurred. By Day 14 post-recanalization, there was mild fluid retention, and a slightly enlarged renal pelvis, and the medulla and cortex layers were visible. 10d: On Day 10 post-obstruction, the left kidney exhibited severe hydronephrosis with marked renal pelvis dilation. By Day 7 post-recanalization, the hydronephrosis had decreased and the renal pelvis was smaller, but the medulla and cortex distinction remained blurred. By Day 14 post-recanalization, there was mild fluid retention and a smaller renal pelvis, but the medulla and cortex layers were still indistinct. 14d: On Day 14 post-obstruction, the left kidney displayed extreme hydronephrosis with significant renal pelvis dilation. By Day 7 post-recanalization, the hydronephrosis had decreased and the renal pelvis was smaller, but the distinction between the medulla and cortex was blurred. By Day 14 post-recanalization, the fluid retention had increased compared to Day 7, the renal pelvis was smaller, and the medulla and cortex boundaries were unclear (Figure 8).

### Dynamic renal scintigraphy of the kidney

**Figure 9.**
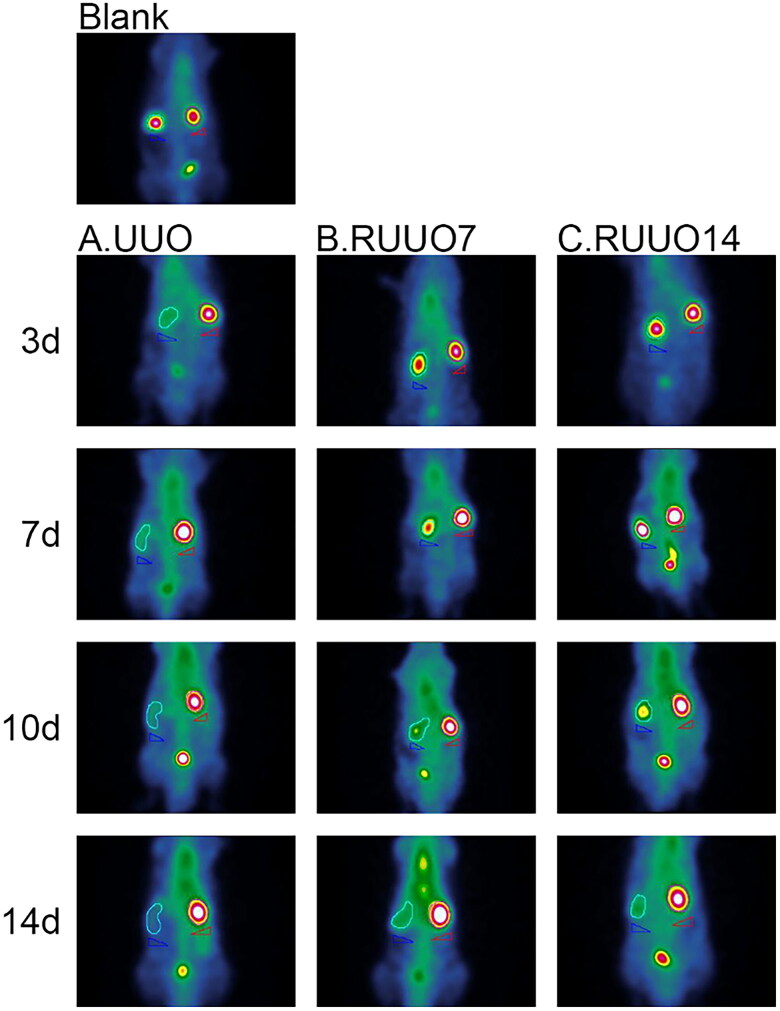
Changes in dynamic renal scintigraphy after obstruction and recanalization. As shown in the figure, the ROI activity in the left kidney decreased with the duration of obstruction. By Day 7 post-recanalization, ROI activity in all groups significantly increased compared with baseline, with the 3-day and 7-day groups showing the most pronounced improvement. By Day 14 post-recanalization, ROI activity was high in all groups except the 14-day group, where it remained low.

Blank: Before left ureteral obstruction, renal scintigraphy showed high activity in the left kidney’s regions of interest (ROIs). The left kidney function was 46.56 (41.10, 59.88) mL/min, and the right kidney function was 49.21 (43.38, 57.60) mL/min. 3d: On Day 3 post-obstruction, renal scintigraphy showed low activity in the left kidney’s ROI, with a GFR of 14.38 (13.81, 16.78) mL/min. On Day 7 post-recanalization, high activity was observed in the left kidney’s ROI, with a significant increase in GFR to 50.39 (42.76, 55.31) mL/min. On Day 14 post-recanalization, high activity persisted in the left kidney’s ROI, and the GFR further increased to 67.26 (64.85, 69.75) mL/min. 7d: On Day 7 post-obstruction, low activity was observed in the left kidney’s ROI, with a GFR of 11.3 (9.675, 18.21) mL/min. On Day 7 post-recanalization, high activity appeared in the left kidney’s ROI, and the GFR improved to 33.47 (21.20, 48.11) mL/min. On Day 14 post-recanalization, high activity was maintained, and the GFR further improved to 54.77 (38.48, 62.39) mL/min. 10d: On Day 10 post-obstruction, the left kidney’s ROI showed low activity, with a GFR of 8.545 (6.27, 10.82) mL/min. On Day 7 post-recanalization, the ROI activity remained low but showed improvement, with a GFR of 18.89 (17.27, 28.46) mL/min. On day 14 post-recanalization, relatively high activity was observed, and the GFR increased to 27.01 (24.99, 35.00) mL/min. 14d: On Day 14 post-obstruction, the left kidney’s ROI showed low activity, with a GFR of 7.46 (2.99, 15.65) mL/min. On Day 7 post-recanalization, the ROI activity improved but remained low, with a GFR of 23.24 (14.99, 34.02) mL/min. On Day 14 post-recanalization, ROI activity declined compared with Day 7, with a GFR of 12.90 (8.905, 23.65) mL/min (Figure 9).

### The results of the left renal function test

**Figure 10.**
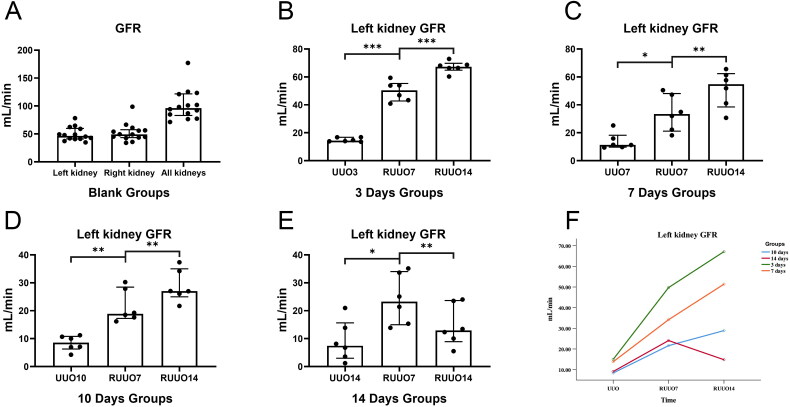
Changes of glomerular rate before and after the release of the obstruction. (A) Left kidney GFR was 46.56 (41.10, 59.88) ml/min; right kidney GFR was 49.21 (43.38, 57.60) ml/min; total kidney GFR was 96.23 (83.01, 121.8) ml/min. (B) Changes in left kidney GFR between UUO3, RUUO7, and RUUO14 were statistically significant (*p* < 0.01). (C) Changes in left kidney GFR between UUO7, RUUO7, and RUUO14 were statistically significant (*p* < 0.05). (D) Changes in left kidney GFR between UUO10, RUUO7, and RUUO14 were statistically significant (*p* < 0.01). (E) Changes in left kidney GFR between UUO14 and RUUO14 were not statistically significant (*p* > 0.05). (F) On Day 7 post-recanalization, the chart indicates that all groups showed significant increases in left kidney GFR. On Day 14 post-recanalization, three groups continued to show increases, although at a slower rate, while the 14days groups exhibited a decline in GFR instead of improvement.

The time effect was statistically significant (*F* = 178.47, *p* = 0.000), indicating that the mean GFR of the left kidney varied across different time points. The interaction between time and treatment was also significant (*F* = 21.96, *p* = 0.000), suggesting that intergroup differences in mean GFR changed over time. Furthermore, intergroup differences in mean GFR were statistically significant (*F* = 25.04, *p* = 0.000; Table 1 and Figure 10).

**Table 1.** Changes in left kidney function across four groups with different obstruction durations and post-ureteral recanalization [median (P25, P75)].

	Left renal GFR (mL/min)				
Groups	UUO	RUUO7	RUUO14	*F* value	*p* value
3 days	14.38 (13.81, 16.78)	50.39 (42.76, 55.31)	67.26 (64.85, 69.75)	231.7	0.000
7 days	11.31 (9.675, 18.21)	33.47 (21.20, 48.11)	54.77 (38.48, 62.39)	35.11	0.000
10 days	8.545 (6.27, 10.82)	18.89 (17.27, 28.46)	27.01 (24.99, 35.00)	61.69	0.000
14 days	7.46 (2.99, 15.65)	23.24 (14.99, 34.02)	12.90 (8.905, 23.65)	11.06	0.002
*F* value	2.60	10.51	49.38	–	–
*p* value	0.091	0.001	0.000	–	–

## Discussion

ON, a common urological condition, requires prompt treatment to avoid renal damage, dysfunction, and failure [15–17]. It is widely recognized that studying the mechanisms of fibrosis and pathophysiological changes in ON require a scientifically sound and practical animal model, which will serve as an essential foundation for such research [18]. The UUO model has become a key animal model for studying ON as it can induce numerous quantifiable pathophysiological events within 1 week [18, 19]. However, the UUO model fails to explain renal injury and repair following the relief of the obstruction. Conversely, the RUUO model offers a clear advantage in this regard. Consequently, a stable, repeatable approach is urgently needed.

Current RUUO models typically use various plastic tubes (silicone [6], elastic plastic [7], and polyethylene [8, 9] or microvascular clamp [10, 11], to wrap or clamp the lateral wall of the ureter, applying an external force to induce obstruction. Recanalization is achieved by removing the plastic pipe days after obstruction. However, these methods have several limitations. First, adhesion between the plastic tube and surrounding adipose tissue often occurs, leading to ureteral tears and urine leakage, as confirmed by Puri et al. [11] and Song et al. [14] These adhesions can compromise surgical success. Second, Puri et al. [11] found an inverse linear relationship between ureter clamping time and recanalization success, suggesting that prolonged clamping causes irreversible damage to the ureter. Consequently, the position of the microvascular clamp was intermittently adjusted to enhance the success rate of recanalization. Despite these adjustments, the recanalization success rate remained at only 60%, as reported by Haque et al. [20] This suggests that the ureter is inherently vulnerable and cannot withstand certain physical stressors. Even with the use of different plastic tubes and intermittent clamp adjustments, permanent ureteral stenosis may still occur due to short-term physical injury. In conclusion, while the procedure is simple and repeatable, its effectiveness remains unstable.

In contrast, three studies used microscopic surgery [12–14], differing from the methods described above. The general procedure involved first inserting a guide needle through the lower left wall of the bladder, guiding the ligated ureter into the bladder from the right upper wall, and then drawing it out from the left wall. Next, the ureter’s end was cut, and the ureter was intermittently sutured to the right bladder wall using microsurgical sutures. Finally, the defect on the left bladder wall was repaired. However, Song et al. [14] encountered several practical issues. Upon opening the bladder, they found that the ureteral segment was encased in connective tissue, likely due to injury from the ureteral incision. To address this, they placed a stent into the ureter to prevent short-term adhesion stenosis. However, the recanalization success rate remained at only 77.5%. We believe this approach still has several limitations. First, although the reported successful rate of 77.5% was claimed, it was not rigorous enough to consider specimens without renal pelvic dilatation as successfully recanalized. Furthermore, while the indwelling stent prevented ureteral adhesion stenosis and allowed for short-term urine drainage, prolonged stent placement could lead to complications such as thrombosis or stone formation, potentially causing hydronephrosis [21–23]. Lastly, the risk of infection and mortality could hinder long-term investigations of the RUUO model.

To address the limitations of the aforementioned models, we proposed a more effective approach. In our study, we completely excised the ligated segment of the ureter during the initial procedure to avoid factors that contribute to ureteral adhesion and stenosis after recanalization—critical elements in achieving high recanalization rates. Additionally, we identified the formation of a wider anastomosis as essential in preventing and managing anastomotic stenosis. This factor significantly improved the success rate of the obstruction recanalization. With our optimized RUUO model, the recanalization success rate reached 93.35%. However, two rats in our experiment exhibited incomplete recanalization, with autopsy revealing necrosis of the ureteral wall at the anastomotic site. This likely resulted from excessive clamping during surgery, which led to insufficient blood supply and necrosis. To avoid such complications, it is essential to minimize traction when isolating the ureter and avoid over-clamping during anastomosis. Additionally, careful and consistent needle insertion and withdrawal are crucial for optimal recovery at the anastomotic site and maintaining a high recanalization success rate.

MRI imaging revealed that hydronephrosis was alleviated, with no renal pelvic dilatation observed during the first week after obstruction release. Furthermore,^99m^Tc-DTPA dynamic renal scintigraphy demonstrated varying degrees of renal function recovery across all groups. In the 3-day obstruction group, renal function returned to normal levels within 1 week post-recanalization. In the 7-day obstruction group, left kidney function recovered to 72% of baseline levels within 1 week. The 10-day obstruction group exhibited a recovery of 40%, while the 14-day obstruction group showed an initial recovery of 50%, followed by a significant decline to 28% after 2 weeks. This study found that the GFR of the left kidney in all groups decreased substantially after obstruction, consistent with expected ureteral obstruction patterns. However, recovery rates varied based on the duration of obstruction. Rats with 3- or 7-day obstructions effective recovery following recanalization, while those with 10- or 14-day obstructions showed limited recovery. In the 14-day UUO group, renal function deteriorated a further 2 weeks after recanalization. We conclude that although GFR is initially maintained following hydronephrosis relief, eventually profound loss of function occurs in the post-obstructed kidney caused by progressive tubulointerstitial and glomerular damage. This is consistent with the conclusions Chevalier et al. [24] and Chan et al. [25] They found that the obstructed renal tissue was not completely repaired at 14 days post-RUUO, and collagen accumulation and extracellular matrix (ECM) imbalance continued, resulting in renal tubule and interstitial fibrosis, glomerular necrosis, sclerosis, and atrophy, ultimately, leading to a decline in filtration function.

A limitation of this study is the direct removal of the bladder valve during surgery, which eliminates the ureterovesical junction mechanism that prevents urine reflux into the ureter. Consequently, urine may accumulate in the ureter when the bladder fills. Although anti-reflux techniques such as the Cohen method [26], the bladder wall loop method [27], the Lich-Gregoir technique [28], the extravesical tunnel method [29], and ureterovesical anastomosis with submucosal tunneling [30] have demonstrated reliable anti-reflux effects, these procedures are complex and challenging to implement practically. Second, a certain level of microsurgical expertise is required, typically necessitating at least three weeks of microsurgical training to establish a stable microsurgical model.

To address these challenges, we developed the RUUO model by completely removing the stricture and directly anastomosing the expanded ureter to a larger bladder opening. This approach offers greater simplicity, higher recanalization success rates, and improved consistency, making it more suitable for researching ON. In the future, we aim to provide a reliable methodology through this integrated approach and detailed experimental design.

## Data Availability

All data generated or analyzed during this study are included in this article. Further inquiries can be directed to the corresponding author.
